# Supplementary material for: Automated detection of COVID-19 through convolutional neural network using chest x-ray images
Source: PLoS One. 2022 Jan 21;17(1):e0262052. doi: 10.1371/journal.pone.0262052 (PMC8782355; doi:10.1371/journal.pone.0262052)
Supplement: Attachment — Submitted filename: Response to reviewers comment.pdf [file pone.0262052.s001.pdf]

Dear Editor,

We greatly appreciate your feedback on how to improve the quality of our manuscript ‘Automated Detection of COVID-19 through Convolutional Neural Network using Chest x-ray Images.” Please find below the point-by-point responses to any concerns raised by the reviewers. The required modifications have been made in the article as suggested. Thank you once again for your constructive comments and opportunity to resubmit again.

Best regards

Comments:

1. Please ensure that your manuscript meets PLOS ONE's style requirements, including those for file naming. The PLOS ONE style templates can be found at https://journals.plos.org/plosone/s/file?id=wjVg/PLOSOne_formatting_sample_main_body.pdf and https://journals.plos.org/plosone/s/file?id=ba62/PLOSOne_formatting_sample_title_authors_affiliations.pdf

Answer to the comment- Thank you for your valuable feedback. We have used PLOS ONE style tamplates to meet the PLOS ONE’s style requirements.

2. Thank you for stating the following financial disclosure:

"The funders had no role in study design, data collection and analysis, decision to publish, or preparation of the manuscript."

At this time, please address the following queries:

a) Please clarify the sources of funding (financial or material support) for your study. List the grants or organizations that supported your study, including funding received from your institution.
b) State what role the funders took in the study. If the funders had no role in your study, please state: “The funders had no role in study design, data collection and analysis, decision to publish, or preparation of the manuscript.”
c) If any authors received a salary from any of your funders, please state which authors and which funders.
d) If you did not receive any funding for this study, please state: “The authors received no specific funding for this work.”

Please include your amended statements within your cover letter; we will change the online submission form on your behalf.

Answer to the comment- Thank you for your comment. “The authors received no specific funding for this work”

3. In your Data Availability statement, you have not specified where the minimal data set underlying the results described in your manuscript can be found. PLOS defines a study's

minimal data set as the underlying data used to reach the conclusions drawn in the manuscript and any additional data required to replicate the reported study findings in their entirety. All PLOS journals require that the minimal data set be made fully available. For more information about our data policy, please see http://journals.plos.org/plosone/s/data-availability.

"Upon re-submitting your revised manuscript, please upload your study's minimal underlying data set as either Supporting Information files or to a stable, public repository and include the relevant URLs, DOIs, or accession numbers within your revised cover letter. For a list of acceptable repositories, please see http://journals.plos.org/plosone/s/data-availability#loc-recommended-repositories. Any potentially identifying patient information must be fully anonymized.

Important: If there are ethical or legal restrictions to sharing your data publicly, please explain these restrictions in detail. Please see our guidelines for more information on what we consider unacceptable restrictions to publicly sharing data: http://journals.plos.org/plosone/s/data-availability#loc-unacceptable-data-access-restrictions. Note that it is not acceptable for the authors to be the sole named individuals responsible for ensuring data access.

We will update your Data Availability statement to reflect the information you provide in your cover letter.

Answer to the comment- Thank you for your comment. "For this work we have used data from public source. Please find the below link."

1) COVID-19 Image Data Collection: Prospective Predictions Are the Future, Joseph Paul Cohen and Paul Morrison and Lan Dao and Karsten Roth and Tim Q Duong and Marzyeh Ghassemi arXiv:2006.11988, https://github.com/ieee8023/covid-chestxray-dataset, 2020
2) https://www.kaggle.com/paultimothymooney/chest-xray-pneumonia, and https://www.cell.com/cell/fulltext/S0092-8674(18)30154-5

4. Please review your reference list to ensure that it is complete and correct. If you have cited papers that have been retracted, please include the rationale for doing so in the manuscript text, or remove these references and replace them with relevant current references. Any changes to the reference list should be mentioned in the rebuttal letter that accompanies your revised manuscript. If you need to cite a retracted article, indicate the article's retracted status in the References list and also include a citation and full reference for the retraction notice.

Answer to the comment- Thank you for your comment. We have made amendments to our reference according to the above instruction.

Added Reference (Most of the reference was from 2020, so we added most cited recent publication from 2021)	1. [35]Hussain, E., Hasan, M., Rahman, M. A., Lee, I., Tamanna, T., & Parvez, M. Z. (2021). CoroDet: A deep learning based classification for COVID-19 detection using chest X-ray images. *Chaos, Solitons & Fractals*, *142*, 110495.
	2. [36]Ismael, A. M., & Şengür, A. (2021). Deep learning approaches for COVID-19 detection based on chest X-ray images. *Expert Systems with Applications*, *164*, 114054.

Retracted Article	1. [21]Butt C, Gill J, Chun D, Babu BA. Deep learning system to screen coronavirusdisease 2019 pneumonia. Applied Intelligence. 2020; p.	The Editor in Chief has retracted this article (1) because the data published here was previously presented by a different set of authors at arXiv pre-print server (2) and a large amount of text, some figures and tables have been re-used without appropriate citation or acknowledgment. The pre-print has been published (3). Author Benson Babu agrees to this retraction. Authors Charmine Butt, Jagpal Gill and David Chun did not respond to any correspondence regarding this retraction. 1) Butt, C., Gill, J., Chun, D. et al. Deep learning system to screen coronavirus disease 2019 pneumonia. Appl Intell (2020). 10.1007/s10489-020-01714-3. 2) Xu, X., Jiang, X., Ma, C., Du, P., Li, X., Lv, S., Yu, L., Chen, Y., Su, J., Lang, G., Li, Y., Zhao, H., Xu, K., Ruan, L., Wu, W. Deep Learning System to Screen Coronavirus Disease 2019 Pneumonia. arXiv:2002.09334v1 [physics.med-ph]. 3) Xu, X., Jiang, X. et al., A Deep Learning System to Screen Novel Coronavirus Disease 2019 Pneumonia, Elsevier, Engineering, 2019. https://www.sciencedirect.com/science/article/pii/S2095809920301636.
Replaced (We replaced priprint cited paper with published citation)	1. [19]Xu X, Jiang X, Ma C, Du P, Li X, Lv S, et al. others," Deep learning system toscreen coronavirus disease 2019 pneumonia,". arXiv preprint arXiv:200209334.2020;<br>2. [64] Wang S, Kang B, Ma J, Zeng X, Xiao M, Guo J, et al. A deep learning algorithmusing CT	[19] Xu, X., Jiang, X., Ma, C., Du, P., Li, X., Lv, S., ... & Li, L. (2020). A deep learning system to screen novel coronavirus disease 2019 pneumonia. *Engineering*, *6*(10), 1122-1129.<br><br>[63] Wang, S., Kang, B., Ma, J., Zeng, X., Xiao, M., Guo, J., ... & Xu, B. (2021). A deep learning algorithm using CT images to screen for Corona Virus Disease (COVID-19). *European radiology*, 1-9.<br><br>[62] Song, Y., Zheng, S., Li, L., Zhang, X., Zhang, X., Huang, Z., ...

		images to screen for Corona Virus Disease (COVID-19). MedRxiv.2020;.<br>3. [62] Song Y, Zheng S, Li L, Zhang X, Zhang X, Huang Z, et al. Deep learning enablesaccurate diagnosis of novel coronavirus (COVID-19) with CT images. medRxiv.2020;.<br>4. [42] Ioffe, S., & Szegedy, C. (2015, June). Batch normalization: Accelerating deep network training by reducing internal covariate shift. In *International conference on machine learning* (pp. 448-456). PMLR.<br>5. [31] Luz E, Silva P, Silva R, Silva L, Guimar˜aes J, Miozzo G, et al. Towards aneffective and efficient deep learning model for COVID-19 patterns detection inX-ray images. Research on Biomedical Engineering. 2021; p. 1–14<br>6. [30] Afshar P, Heidarian S, Naderkhani F, Oikonomou A, Plataniotis KN,Mohammadi	& Yang, Y. (2021). Deep learning enables accurate diagnosis of novel coronavirus (COVID-19) with CT images. *IEEE/ACM Transactions on Computational Biology and Bioinformatics*.<br><br>[42] Ioffe, S., & Szegedy, C. (2015, June). Batch normalization: Accelerating deep network training by reducing internal covariate shift. In *International conference on machine learning* (pp. 448-456). PMLR.<br><br>[31] Luz, E., Silva, P., Silva, R., Silva, L., Guimarães, J., Miozzo, G., ... & Menotti, D. (2021). Towards an effective and efficient deep learning model for COVID-19 patterns detection in X-ray images. *Research on Biomedical Engineering*, 1-14.<br><br>[30] Afshar, P., Heidarian, S., Naderkhani, F., Oikonomou, A., Plataniotis, K. N., & Mohammadi, A. (2020). Covid-caps: A capsule network-based framework for identification of covid-19 cases from x-ray images. *Pattern Recognition Letters*, *138*, 638-643.<br><br>[29] Minaee, S., Kafieh, R., Sonka, M., Yazdani, S., & Soufi, G. J. (2020). Deep-covid: Predicting covid-19 from chest x-ray images using deep transfer learning. *Medical image analysis*, *65*, 101794.<br><br>[24] Li, X., Zhou, Y., Du, P., Lang, G., Xu, M., & Wu, W. (2021). A deep learning system that generates quantitative CT reports for diagnosing pulmonary tuberculosis. *Applied Intelligence*, *51*(6), 4082-4093.<br><br>[9] Wang, L., Lin, Z. Q., & Wong, A. (2020). Covid-net: A tailored

		A. Covid-caps: A capsule network-based framework for identificationof covid-19 cases from x-ray images. arXiv preprint arXiv:200402696. 2020;.<br>7. [29] Minaee S, Kafieh R, Sonka M, Yazdani S, Soufi GJ. Deep-covid: Predictingcovid-19 from chest x-ray images using deep transfer learning. arXiv preprintarXiv:2004 09363. 2020;<br>8. [24] Wu W, Li X, Du P, Lang G, Xu M, Xu K, et al. A Deep Learning System ThatGenerates Quantitative CT Reports for Diagnosing Pulmonary Tuberculosis.arXiv preprint arXiv:191002285. 2019;.<br>9. [9] Wang L, Wong A. COVID-Net: A tailored deep convolutional neural networkdesign for detection of COVID-19 cases from chest radiography images. arXivpreprint arXiv:200309871. 2020;.	deep convolutional neural network design for detection of covid-19 cases from chest x-ray images. *Scientific Reports*, *10*(1), 1-12.<br><br>[8] Narin, A., Kaya, C., & Pamuk, Z. (2021). Automatic detection of coronavirus disease (covid-19) using x-ray images and deep convolutional neural networks. *Pattern Analysis and Applications*, 1-14.

	10. [8] Narin A, Kaya C, Pamuk Z. Automatic detection of coronavirus disease(covid-19) using x-ray images and deep convolutional neural networks. arXivpreprint arXiv:200310849. 2020;.	

5. Please note that in order to use the direct billing option the corresponding author must be affiliated with the chosen institute. Please either amend your manuscript to change the affiliation or corresponding author, or email us at plosone@plos.org with a request to remove this option.

Additional Editor Comments:

Please Address the reviewers' questions one by one.	Thank you for your comment. "We have addressed the reviewers' questions one by one"
The revised manuscript must be the proof-read.	Thank you for your feedback. "Manuscript has gone through proof-read."

Reviewers' comments:

Reviewer #1

Section	Page/Sub-Section	Line	Comment
	P 21. Sec 5	L577	This paper aims to develop a deep learning-based system for the persuasive classification and reliable detection of COVID-19 using chest radiography. Authors evaluate the performance of various state-of-the-art convolutional neural networks (CNNs) proposed over recent years for medical image classification. They have developed and trained CNN from scratch. A recently published public X-Ray dataset for training and validation purposes is used. The comprehensive ROC and confusion metric analysis with 10-fold cross-validation strongly demonstrate the ideas and methods developed in the paper. Experiment results are compared to demonstrate the effectiveness of models in classification scenarios and their potential for COVID-19 classification, detection, prevention, and control.

			The paper is well organised with a clear motivation, innovation and also rich experiments. One minor suggestion is to have two paragraphs in the final section for conclusion and future work.<br><br>- Thank you for your valuable feedback. We further expanded the final sections in two paragraphs.

Reviewer #2

Section	Page/Sub-Section	Line	Comment
	P 2	L40-57	In this manuscript, authors have proposed and presented a CNN oriented COVID-19 detection method, which combine transfer learning and CNN-based model and achieves satisfied results. The idea is interesting. The intention of this study is to evaluate Deep Learning methods for the diagnosis of COVID-19 from X-Ray images. Specifically, both state-of-the-art pretrained CNNs (e.g., VGG) and hand-crafted CNN are utilized either for transfer learning or training from scratch. A relatively small image dataset (at least for the COVID-19 class) is utilized and the results confirm the findings of several related research.<br><br>Nevertheless, there are some issues I would like to point out to enrich the contribution, and the proposal description, which I think must be attended to:<br>• Authors must highlight the contribution in a more profound way to identify the analysis of the several sources consulted.<br><br>-We very much appreciate your feedback. We highlighted the contribution.
	P. 20 Sec. 4.2	L540-541	• Please explain what is CNN II "Therefore, we build a new CNN II which yielded accuracy 97.62% in binary classification" in section 4.2. Probably authors meant Scenario II. Clarify it.<br><br>-Thank you very much for your feedback. We have replaced this sentence with appropriate sentence in "we build a new CNN which yielded accuracy 97.62% in binary classification and 93.75 % in multi-class classification Table 9."
	P14		• In Table 7, the necessary space is missing, which is a little confusing. If possible, rearrange the Table 7.

			-We very much appreciate your comment. We rearranged Table 7.
	P18		• More details need to be presented to show the significance of the results presented in Table 8 and 9.<br><br>-Thank you. We added more details to present the significance of the results presented in Table 8 and 9.
			• Finally, there are some grammatical and structural errors that need to be rectified in the revised version of this manuscript.<br>-Thank you for your valueable comments. We have corrected grammatical and structural errors.

6. PLOS authors have the option to publish the peer review history of their article (what does this mean?). If published, this will include your full peer review and any attached files.

NO
If you choose "no", your identity will remain anonymous but your review may still be made public.

Do you want your identity to be public for this peer review? For information about this choice, including consent withdrawal, please see our Privacy Policy.

Reviewer #1: **Yes:** Jianming Yong

Reviewer #2: No
